# Relationship between nephrotoxicity and area under the concentration–time curve of vancomycin in critically ill patients: a multicenter retrospective study

**DOI:** 10.1128/spectrum.03739-23

**Published:** 2024-05-22

**Authors:** Tomoyuki Ishigo, Kazuaki Matsumoto, Hiroaki Yoshida, Hiroaki Tanaka, Yuta Ibe, Satoshi Fujii, Masahide Fukudo, Hisato Fujihara, Fumihiro Yamaguchi, Fumiya Ebihara, Takumi Maruyama, Yukihiro Hamada, Masaru Samura, Fumio Nagumoi, Toshiaki Komatsu, Atsushi Tomizawa, Akitoshi Takuma, Hiroaki Chiba, Yoshifumi Nishi, Yuki Enoki, Kazuaki Taguchi, Ayako Suzuki

**Affiliations:** 1 Department of Pharmacy, Sapporo Medical University Hospital, Sapporo, Japan; 2 Division of Pharmacodynamics, Keio University Faculty of Pharmacy, Tokyo, Japan; 3 Department of Pharmacy, Kyorin University Hospital, Mitaka, Japan; 4 Department of Pharmacy, Showa University Fujigaoka Hospital, Yokohama, Japan; 5 Department of Hospital Pharmaceutics, School of Pharmacy, Showa University, Tokyo, Japan; 6 Department of Respiratory Medicine, Showa University Fujigaoka Hospital, Yokohama, Japan; 7 Department of Pharmacy, Tokyo Women’s Medical University Hospital, Tokyo, Japan; 8 Department of Pharmacy, Kochi Medical School Hospital, Kochi, Japan; 9 Department of Pharmacy, Yokohama General Hospital, Yokohama, Japan; 10 Department of Pharmacy, Kitasato University Hospital, Sagamihara, Japan; 11 Department of Pharmacy, Showa University Northern Yokohama Hospital, Yokohama, Japan; 12 Department of Pharmacy, Tohoku Kosai Hospital, Sendai, Japan; 13 Center for Pharmacist Education, School of Pharmacy, Nihon University, Funabashi, Japan; University of Pretoria, Pretoria, Gauteng, South Africa

**Keywords:** vancomycin, therapeutic drug monitoring, nephrotoxicity, area under the concentration–time curve

## Abstract

We aimed to assess the frequency of acute kidney injury (AKI) in different areas under the concentration–time curve (AUC) values of vancomycin (VAN) using a two-point blood collection method, allowing for accurate AUC assessment in critically ill patients. This multicenter retrospective observational study was conducted in eight hospitals. We retrospectively analyzed the data of patients who had received VAN in an intensive care unit (ICU) between January 2020 and December 2022. The primary outcome was the incidence of AKI. Patients were classified into three groups according to the AUC_24–48h_ at the initial therapeutic drug monitoring (TDM) as follows: <500, 500–600, and ≥600 µg·h/mL. The AUC_24–48h_ values were calculated using the Bayesian estimation software Practical AUC-guided TDM. Among 146 patients [median age (interquartile range), 67 (56–78) years; 39% women], the AUC_24–48h_ <500 µg·h/mL had an AKI rate of 6.5% (7/107), the AUC_24–48h_ 500–600 µg·h/mL had an AKI rate of 28.0% (7/25), and the AUC_24–48h_ ≥600 µg·h/mL had an AKI rate of 42.9% (6/14). In multivariate Cox proportional hazard analysis, the AUC_24–48h_ 500–600 µg·h/mL [hazard ratio 5.4, 95% confidence interval (CI) 1.64–17.63] and the AUC_24–48h_ ≥600 μg·h/mL (hazard ratio 7.0, 95% CI 2.31–21.18) significantly correlated with a higher incidence of AKI compared with the AUC_24-48h_ <500 μg·h/mL. In conclusion, we identified an association between AUC on day 2 and the risk of AKI in ICU patients, suggesting that not only AUCs above 600 µg·h/mL but also those between 500 and 600 µg·h/mL pose a risk for AKI.

**IMPORTANCE:**

Vancomycin (VAN) is a glycopeptide antibiotic and one of the most commonly used antibiotics for severe infections caused by methicillin-resistant *Staphylococcus aureus*. However, higher VAN concentrations have been associated with an increased risk of acute kidney injury (AKI). Herein, we aimed to assess the frequency of AKI in different areas under the concentration–time curve (AUC) values of VAN using a two-point blood collection method, allowing for accurate AUC assessment in critically ill patients. We identified an association between AUC on day 2 and the risk of AKI in intensive care unit patients, suggesting that not only AUCs above 600 µg·h/mL but also those between 500 and 600 µg·h/mL pose a risk for AKI. Therefore, individualized dosing is feasible, with pharmacists being able to optimize VAN doses to attain appropriate targets.

## INTRODUCTION

Vancomycin (VAN) is a glycopeptide antibiotic and one of the most commonly used antibiotics for severe infections caused by methicillin-resistant *Staphylococcus aureus* (MRSA) (1, 2). Based on pharmacodynamic considerations, the area under the serum concentration–time curve (AUC) divided by the minimum inhibitory concentration (MIC) is the preferred parameter for therapeutic monitoring (3, 4). Regarding efficacy, maintaining the AUC/MIC value above 400 µg·h/mL is recommended (4–6). However, reports indicated a correlation between higher VAN concentrations and an increased risk of acute kidney injury (AKI) (7–9). The risk of AKI increases when VAN trough values exceed 15 or 20 µg/mL (10, 11), which conforms with the findings from meta-analyses (5, 12). AUC-guided monitoring significantly reduces the incidence of AKI compared to that associated with trough-guided monitoring (5, 9, 13, 14). Therefore, recent guidelines recommend dosing designs indexed by the AUC instead of trough values (15, 16). AUC values higher than 1,300 (8), 600 (11, 17, 18), 563 (19, 20), and 550 µg·h/mL (21) have been associated with an increased risk of AKI. Zasowski et al. (22) reported that AUC values of 677 µg·h/mL or higher on day 1 and 683 µg·h/mL or higher on day 2 are associated with an increased risk of AKI. Another report indicated that an AUC of 515 µg·h/mL or higher on day 2 is associated with an increased risk of AKI (23). Meta-analysis identified values higher than 600 (5) or 650 µg·h/mL (24) as indicators for AKI risk. Although high AUC values are demonstrably associated with AKI, reports on the association between early intermediate AUC values and AKI are limited. Consequently, it remains unclear whether an early AUC ranging from 500 to 600 µg·h/mL can be considered as an area associated with AKI. Therefore, in clinical practice, the VAN dosage must be carefully adjusted, even within the reference range, considering both its effectiveness and safety. Particularly, in cases requiring intensive care, there is often a need to administer an adequate amount of VAN for treatment, despite the high risk of AKI. Therefore, determining the frequency of AKI based on AUC values would provide valuable information for the use of VAN in critically ill patients. This study aimed to assess the frequency of AKI for different AUC values using a two-point blood collection method, allowing accurate AUC assessment.

## RESULTS

### Baseline clinical characteristics

**Fig 1:**
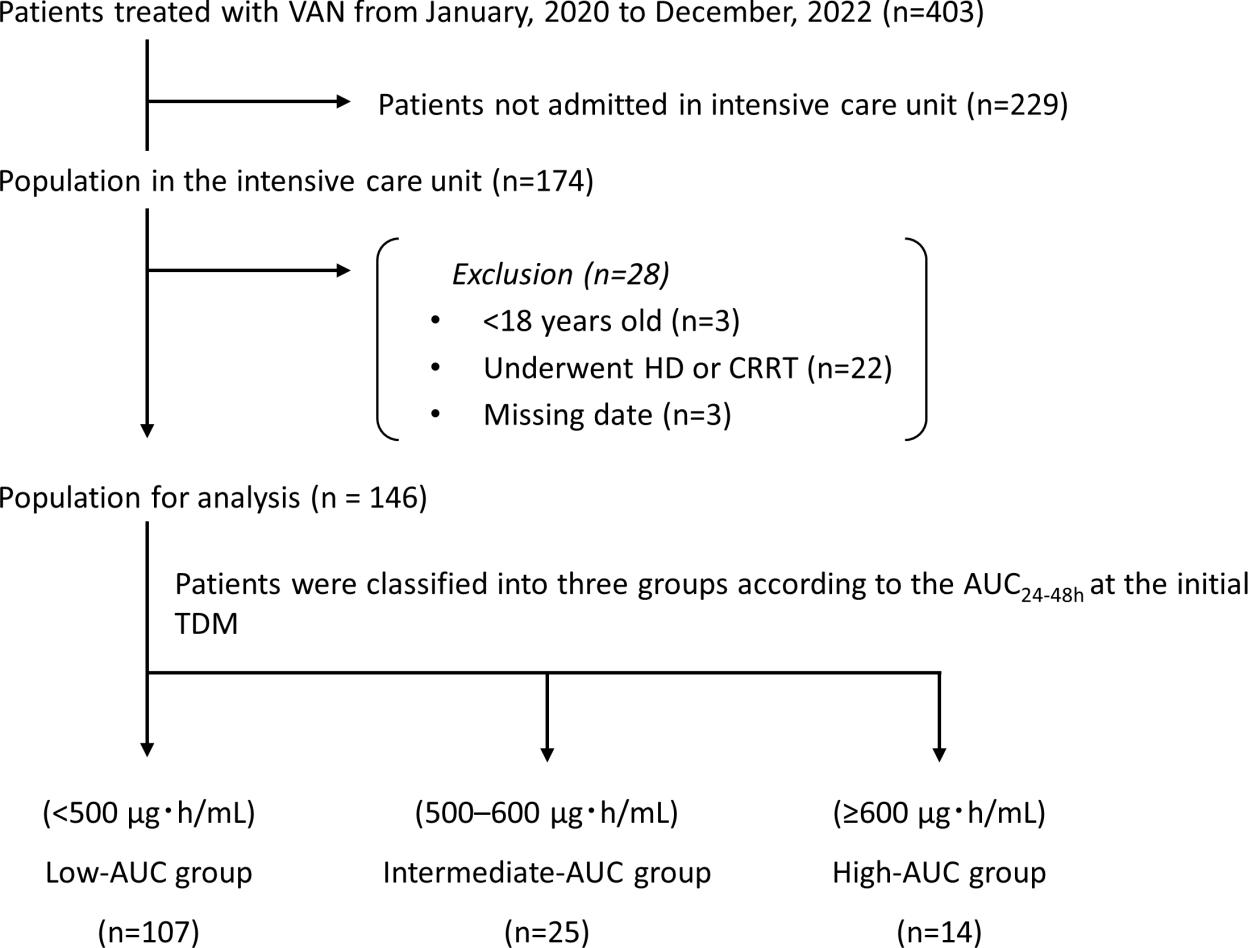
Patient inclusion flowchart for the inclusion of study participants. Abbreviations: VAN, vancomycin; HD, hemodialysis; CRRT, continuous renal replacement therapy; AUC, area under the concentration–time curve; AUC_24–48h_, AUC on day 2; TDM, therapeutic drug monitoring.

This study included 146 patients after applying the exclusion criteria (Fig. 1). The patients had a median age of 67 years [interquartile range (IQR): 56–78 years], and 39% of the patients were female (Table 1). The median body weight and body mass index (BMI) were 60 kg (IQR: 50–69 kg) and 22.3 kg/m^2^ (IQR: 19.3–25.5 kg/m^2^), respectively. The median sequential organ failure assessment (SOFA) and acute physiology and chronic health evaluation II (APACHE II) scores were 6 (IQR: 2–9) and 18 (IQR: 15–24), respectively. Fifty-five (38%) patients had sepsis, 34 (23%) had septic shock, and 64 (44%) had bacteremia (Table 2). Of the 146 patients, 107 had an AUC_24–48h_ at initial TDM of less than 500 µg·h/mL, 25 had an AUC_24–48h_ of between 500 and 600 µg·h/mL, and 14 had an AUC_24–48h_ of greater than 600 µg·h/mL. There were no significant differences in age, sex, SOFA score, APACHE II score, or renal function among the three groups (Table 1). In terms of VAN doses, the loading dose was not significantly different among the three groups; however, the maintenance dose was significantly different, with a trend toward higher doses in the low-, intermediate-, and high-AUC groups (Table 1). There was a significant difference in the AUC_24–48h_ at the dosing design among the three groups, with the intermediate- and high-AUC groups having higher values than those of the low-AUC group (Fig. S1; Table S1).

**TABLE 1:** Patient characteristics^*a*^

	All (*n* = 146)	Low-AUC group (*n* = 107)	Intermediate-AUC group (*n* = 25)	High-AUC group (*n* = 14)	*P* value
Age, years	67 (56, 78)	68 (56, 79)	63 (53, 79)	67 (52, 74)	0.831
Female, n (%)	57 (39%)	39 (36%)	10 (40%)	8 (57%)	0.326
Ht, cm	165 (155, 170)	165 (155, 170)	165 (158, 170)	158 (150, 167)	0.116
Wt, kg	60 (50, 69)	60 (50, 69)	59 (50, 69)	53 (47, 65)	0.650
BMI, kg/m^2^	22.3 (19.3, 25.5)	22.4 (19.7, 25.5)	20.9 (18.6, 24.8)	21.9 (19.8, 25.5)	0.822
SOFA score, *n* = 123	6 (2, 9)	6 (3, 9)	6 (3, 11)	5 (2, 9)	0.711
APACHE II score, *n* = 76	18 (15, 24)	19 (16, 25)	17 (14, 24)	16 (11, 20)	0.253
Laboratory data					
Albumin, g/dL	2.4 (2.0, 2.9)	2.4 (2.0, 2.9)	2.2 (1.8, 2.9)	2.4 (1.7, 2.9)	0.451
Creatinine, mg/dL	0.77 (0.57, 1.08)	0.77 (0.57, 1.06)	0.78 (0.58, 1.24)	0.67 (0.46, 1.26)	0.747
Creatinine <0.6 mg/dL, n (%)	43 (29%)	29 (27%)	7 (28%)	7 (50%)	0.207
CCr, mL/min	72.8 (49.6, 104.3)	73.2 (49.6, 104.7)	62.4 (46.4, 99.6)	71.1 (49.6, 112.0)	0.905
eGFRcre, mL/min/1.73 m^2^	71.6 (47.6, 97.8)	70.9 (48.6, 97.4)	61.9 (43.0, 94.9)	84.2 (46.0, 104.8)	0.821
eGFRcre, <60 mL/min/1.73 m^2^, n (%)	56 (38%)	40 (37%)	11 (44%)	5 (36%)	0.810
eGFRcre, <30 mL/min/1.73 m^2^, n (%)	13 (9%)	12 (11%)	0 (0%)	1 (7%)	0.218
BUN, mg/dL	23 (17, 31)	22 (17, 30)	24 (20, 35)	24 (19, 35)	0.580
BUN/Scr	28.6 (19.7, 42.4)	27.7 (18.9, 39.8)	30.4 (21.5, 43.0)	25.9 (20.2, 61.7)	0.744
Concomitant, n (%)					
Carbapenem	74 (51%)	53 (50%)	13 (52%)	8 (57%)	0.948
TZP	25 (17%)	20 (19%)	1 (4%)	4 (29%)	0.105
Cefepime	10 (7%)	8 (7%)	2 (8%)	0 (0%)	0.747
Aminoglycoside	3 (2%)	1 (1%)	1 (4%)	1 (7%)	0.174
Loop diuretics	54 (37%)	32 (30%)	15 (60%)	7 (50%)	0.011
Catecholamine	69 (47%)	51 (48%)	10 (40%)	8 (57%)	0.582
ACEI/ARB	17 (12%)	15 (14%)	1 (4%)	1 (7%)	0.438
NSAIDs	17 (12%)	12 (11%)	5 (20%)	0 (0%)	0.181
Vancomycin therapy (up to initial TDM)					
Initial dose, mg/kg	25.0 (20.0, 28.7)	24.8 (19.7, 28.0)	26.0 (21.5, 30.0)	26.0 (22.6, 33.5)	0.162
Loading dose (≥25 mg/kg), n (%)	75 (51%)	52 (49%)	16 (64%)	7 (50%)	0.380
Maintenance dose, mg/kg/day	26.0 (20.0, 33.5)	24.4 (18.5, 31.3)	27.8 (21.3, 40.8)	33.3 (24.1, 41.6)	0.003
Dosing interval, n (%)					0.032
8 h	3 (2%)	0 (0%)	1 (4%)	2 (14%)	
12 h	109 (75%)	80 (75%)	19 (76%)	10 (71%)	
24 h	34 (23%)	27 (25%)	5 (20%)	2 (14%)	
Number of doses before initial TDM	2 (2, 3)	2 (2, 3)	2 (2, 4)	2 (2, 4)	0.910
Days to initial TDM, day	3 (2, 3)	3 (2, 3)	2 (2, 3)	2 (2, 3)	0.302
AUC at initial TDM					
AUC_0–24h_, µg·h/mL	429 (335, 503)	384 (322, 461)	509 (446, 567)	525 (458, 660)	<0.001
AUC_24–48h_, µg·h/mL	427 (359, 508)	382 (342, 439)	563 (520, 584)	651 (618, 714)	<0.001
AUC_ss_, µg·h/mL	453 (353, 557)	385 (333, 466)	620 (543, 655)	758 (691, 853)	<0.001

^*a*^ Data are presented as medians (interquartile ranges) or numbers (percentages). Statistical significance was set at *P* < 0.05. Abbreviations: BMI, body mass index; SOFA, sequential organ failure assessment; APACHE II, acute physiology and chronic health evaluation II; CCr, creatinine clearance; eGFRcre, creatinine-based estimated glomerular filtration rate; BUN, blood urea nitrogen; BUN/Scr, ratio of blood urea nitrogen to serum creatinine; TZP, tazobactam/piperacillin; ACEI, angiotensin-converting enzyme inhibitor; ARB, angiotensin receptor blocker; NSAID, non-steroidal anti-inflammatory drug; AUC, area under the concentration–time curve; AUC_0–24h_, AUC on day 1; AUC_24–48h_, AUC on day 2; AUC_SS_, AUC at steady state.

**TABLE 2:** Infectious diseases and causative bacteria^*a*^

	All (*n* = 146)	Low-AUC group (*n* = 107)	Intermediate-AUC group (*n* = 25)	High-AUC group (*n* = 14)	*P* value
Infectious diagnosis, n (%)					
Sepsis	55 (38%)	39 (36%)	10 (40%)	6 (43%)	0.867
Septic shock	34 (23%)	25 (23%)	6 (24%)	3 (21%)	0.983
Bacteremia	64 (44%)	45 (42%)	12 (48%)	7 (50%)	0.767
CRBSI	24 (16%)	15 (14%)	6 (24%)	3 (21%)	0.339
Respiratory tract	24 (16%)	21 (20%)	2 (8%)	1 (7%)	0.314
Skin and soft tissue	19 (13%)	13 (12%)	4 (16%)	2 (14%)	0.778
Surgical site infection	18 (12%)	14 (13%)	2 (8%)	2 (14%)	0.838
Abdomen	17 (12%)	13 (12%)	3 (12%)	1 (7%)	1.000
Meningitis	6 (%)	5 (%)	1 (%)	0 (0%)	1.000
Osteoarticular	3 (2%)	3 (3%)	0 (0%)	0 (0%)	1.000
Infective endocarditis	4 (%)	2 (0%)	0 (0%)	2 (14%)	0.087
Others	14 (10%)	8 (7%)	3 (12%)	3 (21%)	0.188
Unknown	16 (11%)	12 (11%)	4 (16%)	0 (0%)	0.333
Causative bacteria, n (%)					
MRSA	11 (8%)	3 (3%)	5 (20%)	3 (21%)	0.002
MR-CNS	10 (7%)	7 (7%)	1 (4%)	2 (14%)	0.468
*Enterococcus faecium*	5 (3%)	4 (4%)	0 (0%)	1 (7%)	0.544
Others	43 (29%)	34 (32%)	6 (24%)	3 (21%)	0.655

^*a*^ The number of cases included overlaps. Data are presented as numbers (percentages). Statistical significance was set at *P* < 0.05. Abbreviations: CRBSI, catheter-related bloodstream infection; MRSA, methicillin-resistant *Staphylococcus aureus*; MR-CNS, methicillin-resistant coagulase-negative *staphylococci*.

### Relationships of the VAN AUC_24–48h_ level with the frequency of AKI

The primary outcome of AKI was observed in 20 (13.7%) patients. The high-AUC group had an AKI rate of 42.9% (6/14), the intermediate-AUC group had an AKI rate of 28.0% (7/25), and the low-AUC group had an AKI rate of 6.5% (7/107). Patients with an AUC_24–48h_ of ≥500 µg·h/mL had a significantly higher incidence of AKI than that of patients with an AUC_24–48h_ of <500 µg·h/mL (*P* < 0.001). In the low-AUC group, the dose of VAN was increased after the initial TDM in 44 (41%) patients, decreased in 9 (8%), kept at the same dose in 38 (36%), and discontinued in 16 (15%). In the intermediate-AUC group, the dose was reduced after the initial TDM in 15 (60%) patients, kept at the same dose in 4 (16%), and discontinued in 6 (24%). In the high-AUC group, the dose was reduced after the initial TDM in 7 (50%) patients, kept at the same dose in 4 (29%), and discontinued in 3 (21%).

### Validation of factors associated with AKI

The predictive performances of the CART-derived and other toxicity threshold candidates are listed in Table 3. Nephrotoxicity was significantly higher among patients with an AUC_24-48h_ of ≥462 µg·h/mL and AUC_ss_ of ≥651 µg·h/mL, while no threshold was discovered for blood urea nitrogen (BUN)/serum creatinine (Scr) (Table 3; Fig. S2). In the analyses in which all patients were included, an AUC_24–48h_ of 462 µg·h/mL had an accuracy of 72%, a sensitivity of 80%, and a specificity of 71% for the prediction of AKI; that of 500 µg·h/mL had an accuracy of 77%, a sensitivity of 65%, and a specificity of 79% for the prediction of AKI; whereas that of 600 µg·h/mL had an accuracy of 85%, a sensitivity of 30%, and a specificity of 94% for the prediction (Table 3).

**TABLE 3:** Diagnostic accuracy of each factor for AKI^*b*^

	AUC	Cut-off value	Sensitivity	Specificity	PPV	NPV	Accuracy	TP	TN	FP	FN	*P* value
AUC_24–48h_	0.78	400	0.90	0.48	0.21	0.97	0.53	18	60	66	2	<0.001
		462^*a*^	0.80	0.71	0.30	0.96	0.72	16	89	37	4	
		500	0.65	0.79	0.33	0.93	0.77	13	100	26	7	
		600	0.30	0.94	0.43	0.89	0.85	6	118	8	14	
AUC_ss_	0.81	400	0.90	0.45	0.21	0.97	0.51	18	57	69	2	<0.001
		500	0.80	0.71	0.31	0.96	0.73	16	90	36	4	
		600	0.65	0.84	0.41	0.94	0.82	13	107	19	7	
		651^*a*^	0.60	0.90	0.52	0.93	0.87	12	115	11	8	
BUN/Scr	0.52	51.9^*a*^	0.30	0.87	0.26	0.89	0.79	6	109	17	14	0.465

^*a*^ Optimal cut-off values.

^*b*^ Predictive performance of CART-derived and other candidate AUC toxicity thresholds. Abbreviations: AKI, acute kidney injury; AUC, area under the concentration–time curve; AUC_24–48h_, AUC on day 2; AUC_SS_, AUC at steady state; BUN/Scr, ratio of blood urea nitrogen to serum creatinine; PPV, positive predictive value; NPV, negative predictive value; TP, true positive; TN, true negative; FP, false positive; FN, false negative; CART, classification and regression tree.

### Relationship between AUC_24–48h_ level and time to AKI onset

**Fig 2:**
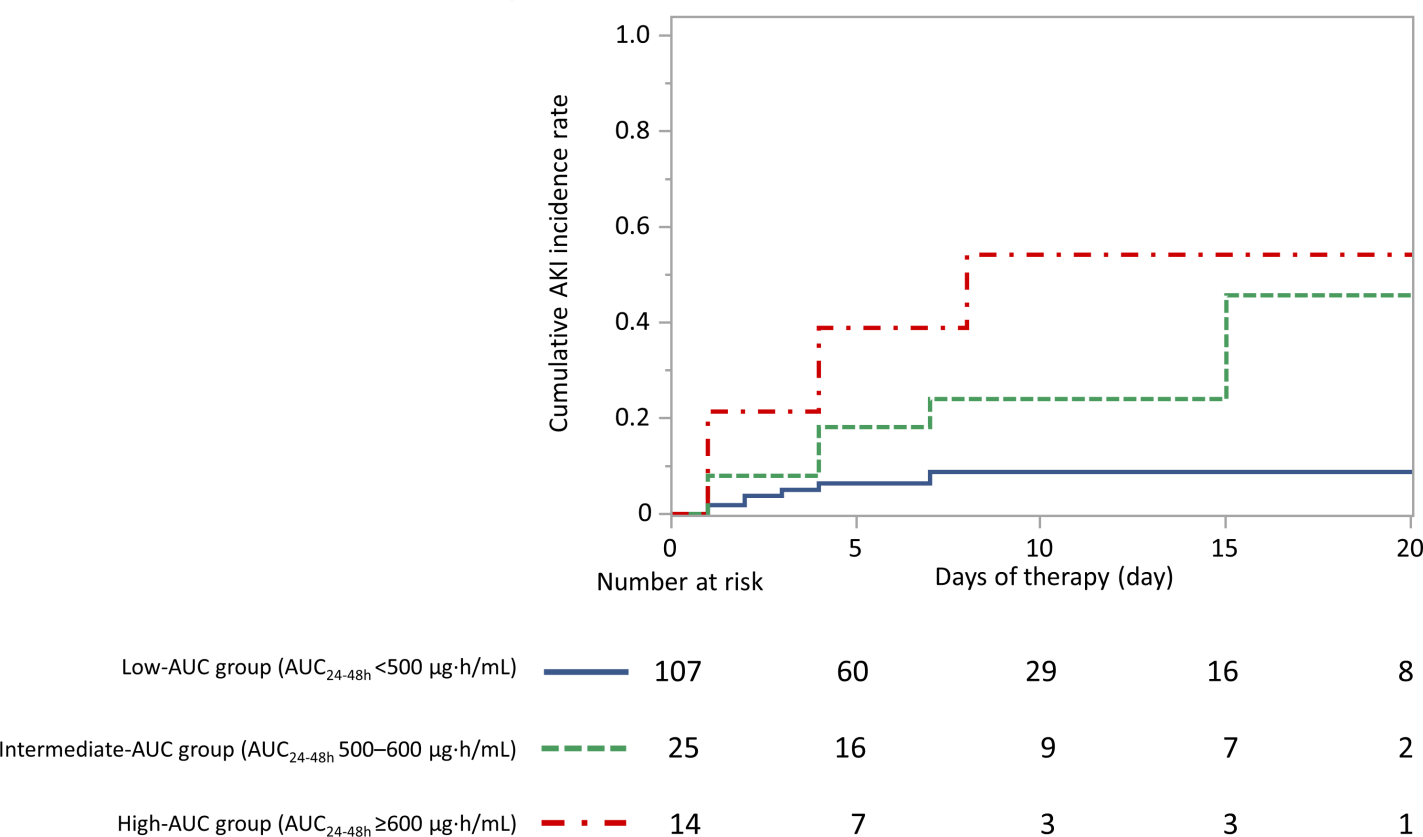
Relationship between the AUC and the cumulative rate of AKI Log-rank: low-AUC vs. intermediate-AUC group, *P* = 0.010; low-AUC vs high-AUC group, *P* < 0.001; intermediate-AUC vs. high-AUC group, *P* = 0.284. To account for the increased alpha error, the Bonferroni correction was applied to the comparison of the three groups, and a *P*-value of less than 0.0167 was considered significant. Abbreviations: AKI, acute kidney injury; AUC, area under the concentration–time curve.

Kaplan–Meier curves revealed that the cumulative rate of AKI was the highest in the high-AUC group (Fig. 2). Compared to those observed in the low-AUC group, the medium- and high-AUC groups had significantly higher AKI rates (log-rank *P* = 0.010 and *P* < 0.001, respectively; Fig. 2).

### Cox proportional hazard analyses for factors predicting AKI onset

In univariate analyses, AUC_24–48h_ {intermediate AUC: hazard ratio [HR], 3.7 [95% confidence interval (CI), 1.29–10.58]; *P* = 0.015; high-AUC: HR, 6.6 [95% CI, 2.20–19.56]; *P* < 0.001} was significantly associated with AKI onset (Table 4). In a multivariate analysis adjusted for serum creatinine-based estimated glomerular filtration rate (eGFRcre) <30 mL/min/1.73 m^2^, the use of tazobactam/piperacillin (TZP), and the use of loop diuretic, which are risk factors for AKI (25), intermediate-AUC [HR, 5.4 (95% CI, 1.64–17.63); *P* = 0.006], high-AUC [HR, 7.0 (95% CI, 2.31–21.18); *P* < 0.001], and the use of TZP [HR, 3.2 (95% CI, 1.05–9.53); *P* = 0.040] were significantly associated with a higher incidence of AKI (Table 4).

**TABLE 4:** Cox proportional hazard analyses of factors associated with AKI^*a*^

	Univariate model			Multivariate model 1			Multivariate model 2		
	HR	(95% CI)	*P* value	HR	(95% CI)	*P* value	HR	(95% CI)	*P* value
Age, per 1-year increase	1.0	0.98–1.05	0.405						
Sex; female	0.8	0.33–2.00	0.652						
BMI, per 1 kg/m^2^ increase	1.0	0.92–1.10	0.704						
SOFA score	1.0	0.92–1.16	0.560						
APACHE II score	1.0	0.91–1.12	0.912						
Sepsis	1.0	0.41–2.45	0.997						
Septic shock	1.8	0.72–4.51	0.212				1.6	0.63–4.28	0.315
VAN AUC_24–48h_									
<500 µg·h/mL	Reference	–	–	Reference	–	–	Reference	–	–
500–600 µg·h/mL	3.7	1.29–10.58	0.015	5.4	1.64–17.63	0.006	4.4	1.46–13.13	0.009
≥600 µg·h/mL	6.6	2.20–19.56	<0.001	7.0	2.31–21.18	<0.001	8.2	2.52–26.86	<0.001
Loding dose, ≥25 mg/kg	1.0	0.40–2.30	0.917				1.1	0.43–2.77	0.863
Maintenance dose, mg/kg/day	1.0	0.96–1.05	0.765				0.98	0.93–1.02	0.290
eGFRcre, <30 mL/min/1.73 m^2^	0.6	0.08–4.37	0.601	1.0	0.12–7.78	0.962			
BUN/Scr, ≥20	1.2	0.38–3.46	0.799						
TZP	2.1	0.79–5.40	0.136	3.2	1.05–9.53	0.040			
Catecholamine	2.1	0.83–5.21	0.119						
Loop diuretic	1.2	0.49–2.88	0.704	0.8	0.32–1.98	0.628			

^*a*^ Statistical significance was set at *P* < 0.05. To account for the increased alpha error, the Bonferroni correction was applied to the comparison of the three groups, and *P*-values less than 0.0167 were considered significant. Abbreviations: AKI, acute kidney injury; HR, hazard ratio; CI, confidence interval; BMI, body mass index; SOFA, sequential organ failure assessment; APACHE II, acute physiology and chronic health evaluation II; VAN, vancomycin; AUC, area under the concentration–time curve; AUC_24–48h_, AUC on day 2; eGFRcre, creatinine-based estimated glomerular filtration rate; BUN/Scr, ratio of blood urea nitrogen to serum creatinine; TZP, tazobactam/piperacillin.

### Association of AUC_24–48h_ with probability of AKI estimated using univariate logistic regression analysis

**Fig 3:**
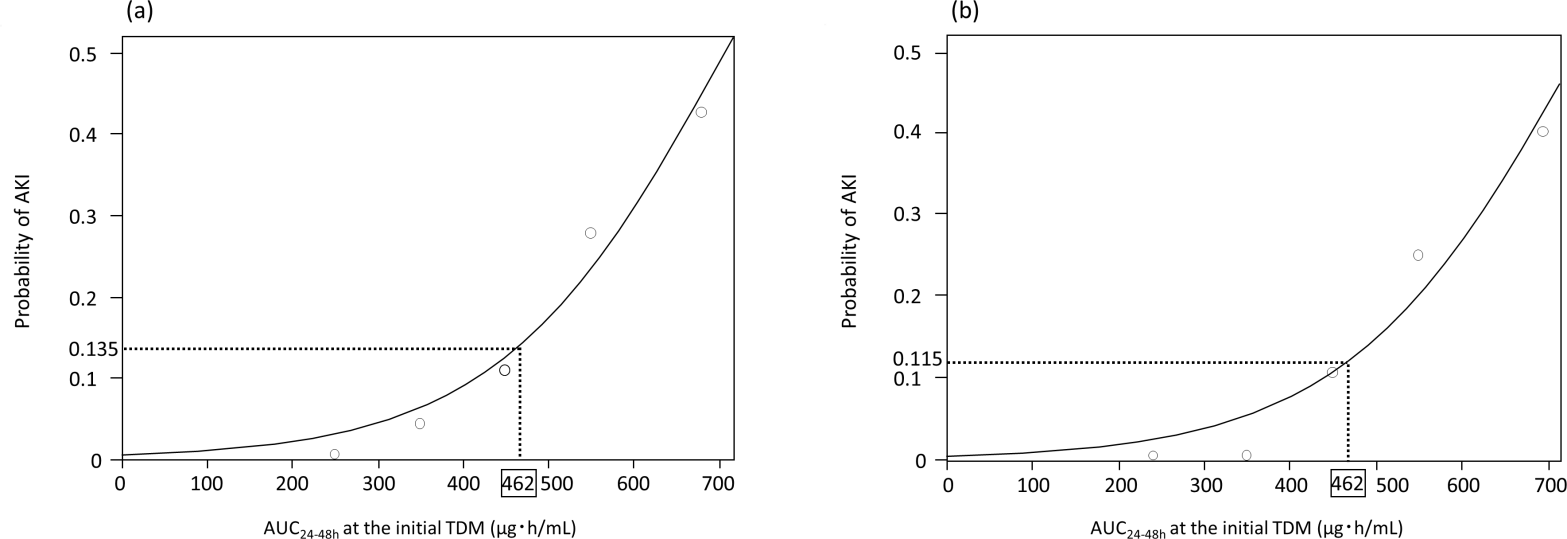
Vancomycin exposure–toxicity curve. The relationship between the AKI risk and the vancomycin AUC_24–48h_ is represented using a best-fit curve. The CART threshold is indicated by the vertical dashed lines. (**A**) All patients (*n* = 146) and (**B**) patients without concomitant TZP (*n* = 121). Abbreviations: AKI, acute kidney injury; AUC, area under the concentration–time curve; TDM, therapeutic drug monitoring; CART, classification and regression tree; TZP, tazobactam/piperacillin.

Among all 146 patients, a univariate logistic regression curve indicated that an AUC_24–48h_ of 462 µg·h/mL was estimated by an AKI probability of 13.5% (Fig. 3a). According to inverse estimation, an AKI probability of 10% was estimated by an AUC_24–48h_ of 417 (95% CI, 355–478) µg·h/mL, and an AKI probability of 15% was estimated by an AUC_24–48h_ of 478 (95% CI, 432–524) µg·h/mL (Table S2; Fig. 3a); this implies a safe target AUC_24–48h_. In an analysis of 121 patients without the use of TZP, the univariate logistic regression curve estimated the AKI probability to be 11.5% when the AUC_24–48h_ was 462 µg·h/mL (Tables S2 and S3; Fig. 3b). According to inverse estimation, an AKI probability of 10% was estimated by an AUC_24–48h_ of 441 (95% CI, 359–522) µg·h/mL, and an AKI probability of 15% was estimated by an AUC_24–48h_ of 504 (95% CI, 442–565) µg·h/mL (Table S2; Fig. 3b); the other values are shown in Table S2.

## DISCUSSIONS

Our results suggest an association between the AUC on day two and the risk of AKI in intensive care unit (ICU) patients, indicating that not only AUCs above 600 µg·h/mL but also those between 500 and 600 µg·h/mL pose a risk for AKI. From the perspective focused on AKI prevention, these results provide novel insights into the previous recommendation of not exceeding a steady-state AUC of 600 µg·h/mL. For the dosing design, an AUC of less than 500 µg·h/mL on day 2 is recommended.

Our study had several strengths: (1) We enrolled patients from eight institutions in Japan, all of which studied ICU patients (2). We calculated the accurate AUC using a two-point blood collection method (3). We conducted additional analysis excluding TZP.

The first strength of this study was that we enrolled ICU patients from eight Japanese centers to determine the association between AUC and the risk of AKI. A systematic review reported a mean range of AKI occurrence of 4.3–17 days (12) after the initiation of VAN, with onset reported as early as 2–3 days after the initiation of therapy (26, 27). The incidence of VAN-induced AKI varies from 5% to 43% (12) depending on various factors such as the definition of AKI, VAN exposure, concomitant use of TZP or diuretics, disease severity, and other potential risk factors. ICU patients (16%–22%) are particularly at a higher risk of AKI than non-ICU patients (3%–7%) (8, 25, 28). Our overall AKI incidence of 13.7% was similar to that of recent large retrospective clinical studies that reported incidences of 11.7% in critically ill patients (29) and 15.8% in critically ill Japanese patients (25). However, some reports have indicated higher AKI frequencies in ICU patients using trough-based dosing designs, such as 20% (8) or 24% (10). In a study of 1,882 patients using VAN, Hashimoto et al. (25) calculated the optimal cutoff values for groups with AKI risk factors (e.g., CKD, concomitant use of TZP, or diuretics). However, in the ICU cases, the optimal cutoff values could not be calculated because of the small number of cases (25). AUC-guided monitoring significantly reduces the incidence of AKI compared to that associated with trough-guided monitoring (5, 9, 13, 14). Therefore, indexing ICU patients by AUC may be useful in reducing the risk of AKI, and the results of this study may provide additional information on AUC cutoff values and AKI frequency in ICU patients. For ICU patients at high risk of AKI, an AUC of 500–600 µg·h/mL on day 2 was found to be associated with an increased risk of AKI. Since the diagnoses of infection and isolated bacteria are important factors in the treatment of VAN, we incorporated factors, such as septic shock, sepsis, and MRSA bacteremia, in addition to VAN AUC and dosage, with similar results (Table 4 and S4). A recent study reported that patients with AUCs between 400 and 550 µg·h/mL rarely experienced AKI (21). Because an early AUC of 400 µg·h/mL or higher has been associated with early efficacy (30), a target of 400–500 µg·h/mL should be considered for efficacy and safety in the initial dosing design. In ICU patients, CART analysis suggested that an AUC_24–48h_ below 462 µg·h/mL may reduce the frequency of AKI (Fig. 3). Depending on the type of isolate (or if the bacteria are undetectable), VAN may be discontinued early, but this study revealed the importance of early AUC, and the initial dosing design of VAN may be important even for early discontinuation.

Second, we calculated the AUC using a two-point blood draw method. Conducted in ICU patients, this study suggests that evaluating the AUC using trough and peak values is significant, considering the effects of circulatory dynamics and volume of distribution (15, 31, 32). Previous AUC studies mostly used AUCs calculated from trough values alone (19, 22, 23). Although trough values can predict AUCs to some extent, the AUCs calculated using two-point blood sampling are clearly more accurate (33). Therefore, we are confident that the AUC values obtained in this study more accurately explain the relationship between AUC and AKI risk. A recent meta-analysis reported a reduced probability of AKI and a higher probability of achieving pharmacokinetic targets when VAN is administered as a continuous infusion compared to the probabilities associated with intermittent infusion without affecting total mortality, suggesting that continuous administration is also an important treatment option (34). However, Japanese guidelines do not recommend continuous VAN administration due to lack of evidence (15). Therefore, in this study, no cases were treated with continuous administration. Continuous administration is an important treatment option, and further accumulation of evidence in Japan is required.

Third, the additional analysis excluded TZP. Although literature regarding TZP-induced AKI is still incomplete, its combination with VAN carries a higher risk of AKI than those associated with other beta-lactams (such as carbapenems and cefepime) (35, 36). Presumably, the concomitant use of TZP is an independent risk factor unrelated to the serum concentrations of VAN (35, 36), and several studies have reported the additive nephrotoxic effects of acute interstitial nephritis and direct cell necrosis (37, 38). This study also revealed that the combination of TZP and VAN was an independent risk factor for AKI regardless of the AUC value. Univariate logistic regression and CART analyses were performed to calculate the probability of AKI in the absence of concomitant TZP. The absence of the TZP combination resulted in a 2% reduction in the frequency of AKI according to the cutoff value indicated for the AUC (Fig. 3) and a wider safe range of AUCs corresponding to the respective AKI frequencies (Table S2). These results support the findings of Oda et al. (39) that the frequency of AKI decreases with interventions that prevent the use of VAN and TZP combination therapy. Further validation of the association between the combination of TZP–VAN and AKI is needed; both TZP and VAN can bind to renal transporters that mediate creatinine secretion (40, 41). Furthermore, a recent study examining changes in creatinine in patients treated with VAN and TZP suggested that the increase in creatinine may be pseudo-nephrotoxicity (42). Animal models suggest that TZP may reduce the nephrotoxicity of VAN (43). Since this study also evaluated creatinine-based AKI, pseudo-nephrotoxicity should be considered. To clarify these problems, the renal injury with cystatin C needs to be evaluated in combination.

Nevertheless, our study had several limitations. First, as this was a retrospective observational study, some selection bias might have occurred in the study population. However, because patient data were extracted from eight different Japanese hospitals, the selection bias was likely smaller than that of a single-hospital study. Second, owing to the limited number of cases, we were unable to validate the probability of AKI using only patients with a combination of TZP and VAN. Third, interpretating these results is difficult because it is unclear whether an elevated AUC increases the risk of AKI or whether AKI increases the likelihood of an elevated AUC (36). However, our results suggest that the intermediate- and high-AUC groups were designed with higher doses than those associated with the low-AUC group from the initial dosing design stage (Table S1), which may explain why an elevated AUC increases the risk of AKI. Fourth, this study did not include cases in which VAN was administered continuously. Fifth, this study did not examine VAN preparation and conservation methods. Sixth, due to the limited number of AKI cases, we could not examine the association between AUC and severity of AKI and subsequent transition to maintenance dialysis. Finally, this study did not evaluate the efficacy of VAN. These aspects should be addressed in future studies.

In conclusion, we found an association between AUC on day 2 and the risk of AKI in ICU patients, suggesting that not only AUCs above 600 µg·h/mL but also those between 500 and 600 µg·h/mL pose a risk for AKI. Furthermore, an AUC of less than 462 µg·h/mL on day 2 may minimize the risk of AKI. We suggest that the initial dosing be designed so that the AUC on day 2 does not exceed 500 µg·h/mL. Our data indicate that individualized dosing is feasible, with pharmacists being able to optimize VAN doses to attain appropriate PK/PD targets. An additional benefit of such an approach is the refinement of the guidelines for MRSA infections, with the respective AUC targets set according to the risk of AKI.

## MATERIALS AND METHODS

### Study participants

This multicenter retrospective observational study was conducted at Sapporo Medical University Hospital, Kyorin University Hospital, Tokyo Women’s Medical University Hospital, Showa University Fujigaoka Hospital, Yokohama General Hospital, Kitasato University Hospital, Showa University Northern Yokohama Hospital, and Tohoku Kosai Hospital. We retrospectively analyzed data from patients who had received at least 24 h of VAN in the ICU from January 2020 to December 2022. The enrollment protocol for this study is shown in Fig. 1. Patients younger than 18 years, those undergoing hemodialysis or continuous renal replacement therapy, and those with missing data for peak or trough VAN were excluded. Patients were classified into three groups according to the AUC_24–48h_ at the initial TDM as follows: the low-AUC group (<500 µg·h/mL), intermediate-AUC group (500–600 µg·h/mL), and the high-AUC group (≥600 µg·h/mL).

### Outcomes

The primary outcome was the incidence of AKI, which was defined according to the Kidney Disease Improving Global Outcomes criteria [i.e., an increase in Scr of ≥0.3 mg/dL (within 48 h), of ≥50% from the most recent pre-treatment data during therapy or continuation of a urine volume of 0.5 mL/kg/h for 6 h or longer] (44). We calculated the sensitivity, specificity, positive predictive value, negative predictive value, and accuracy to evaluate the diagnostic abilities of AUC_24–48h_, AUC_ss_, and BUN/Scr. The pharmacokinetic profiles of VAN were retrospectively analyzed from the trough and peak levels using the Bayesian estimation software Practical AUC-guided TDM (PAT) (ver. 3.0) (33).

### Data collection

Information regarding the patient’s age, sex, body weight, height, BMI, Scr, creatinine clearance (CCr), eGFRcre, VAN dosage, VAN concentrations (trough and peak levels), AUC, infectious diagnosis, causative bacteria, SOFA and APACHE II scores, and concomitant medications was obtained from the patient’s medical records. The absence of bacterial growth after 7 days of appropriate incubation was defined as "absence of blood cultures.” Blood samples were collected immediately (within 30 min) before VAN administration to obtain trough concentrations. Samples for peak concentrations were collected 0.5–4 h after an intravenous VAN infusion. CCr was calculated using the Cockcroft–Gault formula (Eq. 1) (45). The eGFRcre was calculated using an equation provided by the Japanese Society of Nephrology (Eq. 2) (46).


(Eq. 1) $$CCr\;\left(mL/min\right)=\;\frac{\left(140-Age\right)\;\times \;Body\;Weight\;\left(kg\right)}{72\;\times \;Scr\;\left(mg/dL\right)}\;\left(\times 0.85\;if\;female\right)$$

(Eq. 2) $$eGFRcre\;\left(mL/min/1.73\;\;m^{2}\right)=194\;\times Scr^{-1.094}\times Age^{-0.287}\;\left(\times 0.739\;if\;female\right)$$

### Statistical analyses

Data were presented as medians (IQR: 25th–75th percentile) and expressed as frequencies and percentages. Welch’s test was used to compare continuous variables between groups. Differences in categorical variables between the two groups were examined using the chi-squared test and Fisher’s exact test. Patient backgrounds of the three groups divided by AUC_24–48h_ were compared using the Kruskal–Wallis test. A classification and regression tree (CART) analysis was applied to identify the threshold value for the AKI onset day and the AUC_24–48h_ of the VAN cutoff capable of predicting an increased risk of AKI. Receiver operating characteristic curves were used to calculate the area under the curve and cutoff values for predicting AKI. Kaplan–Meier curves and log-rank tests were used to assess the cumulative frequency of AKI. Univariate and multivariate Cox proportional hazard analyses were performed to identify potential factors that may have influenced the clinical outcomes. To account for the increased alpha error, the Bonferroni correction was applied to the comparison of the three groups, and *P*-values less than 0.0167 were considered significant. The software programs JMP Pro 17.0 (SAS Institute Inc., Cary, NC, USA) and EZR version 1.54 (Saitama Medical Center, Jichi Medical University, Saitama, Japan), which is a graphical user interface for R (The R Foundation for Statistical Computing, Vienna, Austria), were used for the statistical analyses in this study.

## Data Availability

The data sets generated and/or analyzed during the current study are not publicly available because a research agreement from all authors is required for data sharing, but are available from the corresponding author on reasonable request.

## SUPPLEMENTAL MATERIAL

Supplemental figures spectrum.03739-23-s0001.pdf Fig. S1 and S2.

Supplemental tables spectrum.03739-23-s0002.docx Tables S1-S4.

ASM does not own the copyrights to Supplemental Material that may be linked to, or accessed through, an article. The authors have granted ASM a non-exclusive, world-wide license to publish the Supplemental Material files. Please contact the corresponding author directly for reuse.
