# Supplementary material for: Relationship between nephrotoxicity and area under the concentration–time curve of vancomycin in critically ill patients: a multicenter retrospective study
Source: Microbiol Spectr. 2024 May 22;12(7):e03739-23. doi: 10.1128/spectrum.03739-23 (PMC11324017; doi:10.1128/spectrum.03739-23)
Supplement: Supplemental figures — Fig. S1 and S2. [file spectrum.03739-23-s0001.pdf]

Figure S1

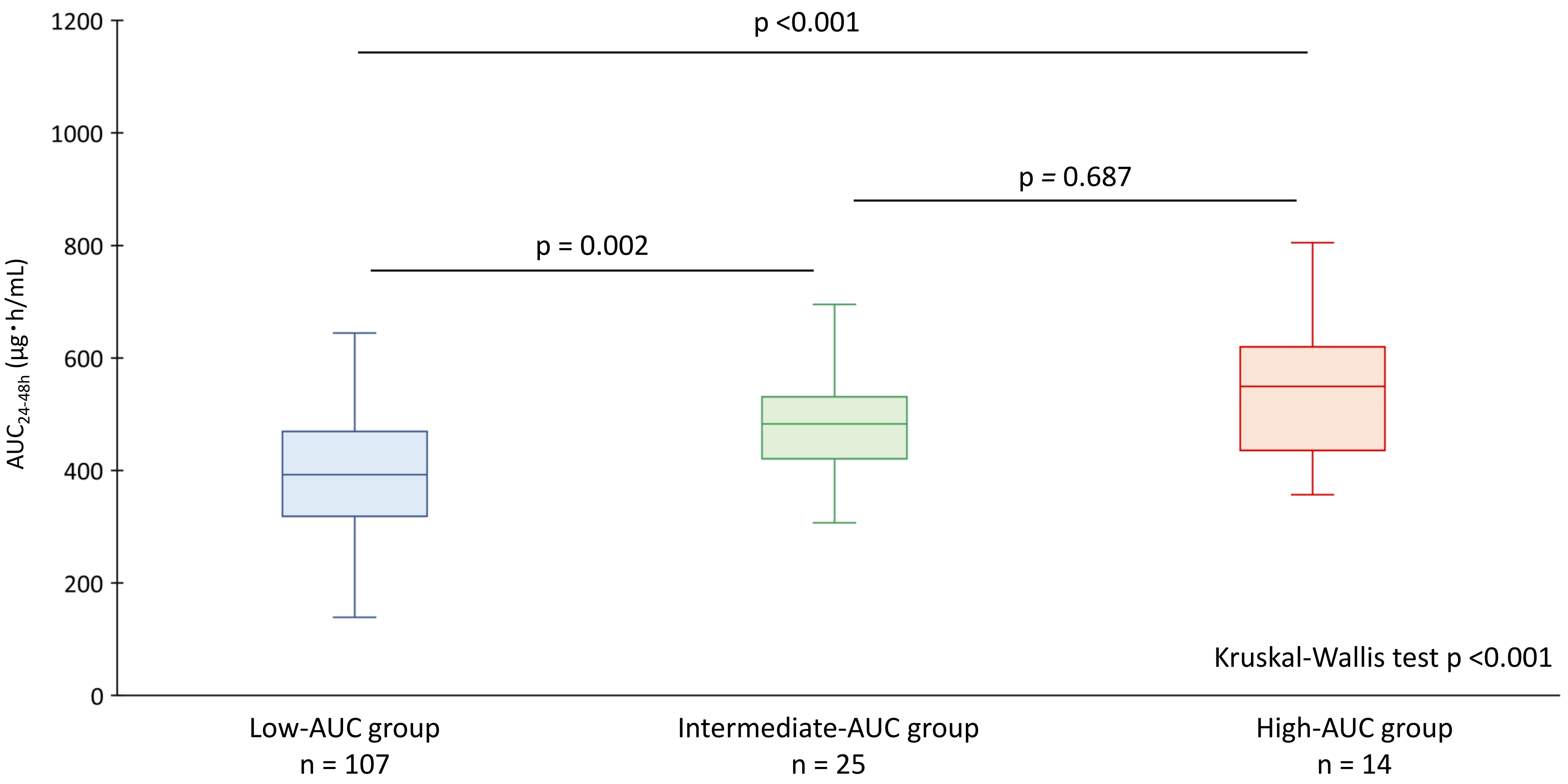

$AUC_{24\text{-}48h}$ at the dosing design among the three groups. Patients were classified into three groups according to the $AUC_{24\text{-}48h}$ at the initial TDM as follows: the low-AUC group (<500 μg·h/mL), intermediate-AUC group (500–600 μg·h/mL), and the high-AUC group (≥600 μg·h/mL). The Kruskal-Wallis test was used to test the overall difference among the three groups, and the Dunn test was used to compare two groups. Bonferroni correction was applied to account for the increase in alpha error.

Figure S2

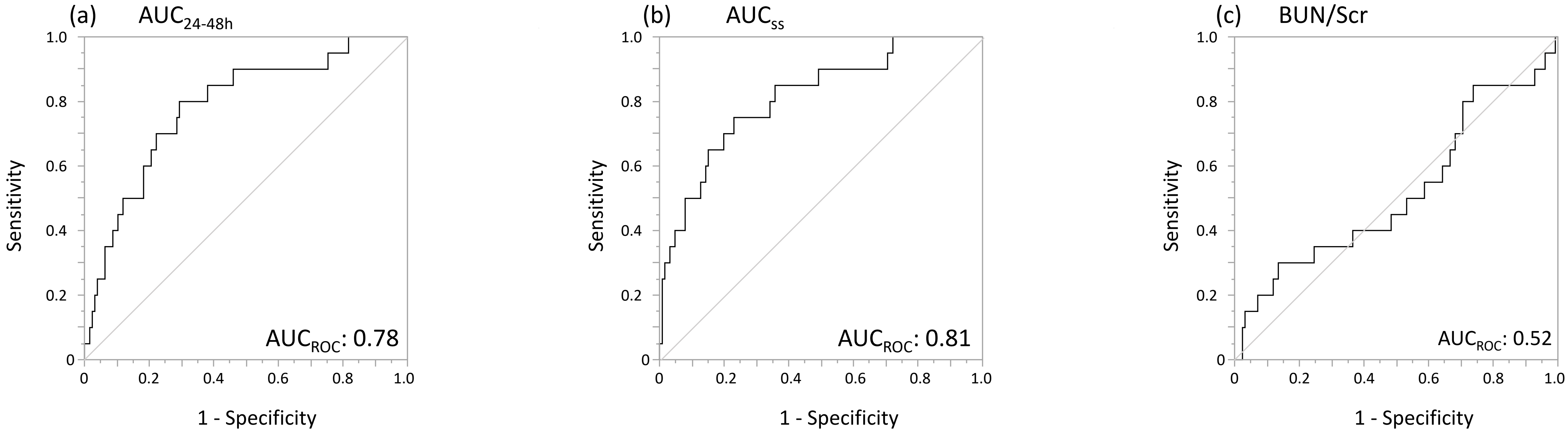

ROC curve for predicted AKI of (a) $AUC_{24\text{-}48h}$, (b) AUC_{ss}, and (c) BUN/Scr. The AUC_{ROC} shown in the figure represents the area under the ROC curve. Abbreviations: ROC, receiver operating characteristic; AKI, acute kidney injury; AUC, area under the concentration-time curve; BUN/Scr, ratio of blood urea nitrogen to serum creatinine; $AUC_{24\text{-}48h}$, AUC on day 2; AUC_{ss}, AUC at steady-state.
