# Supplementary material for: Relationship between nephrotoxicity and area under the concentration–time curve of vancomycin in critically ill patients: a multicenter retrospective study
Source: Microbiol Spectr. 2024 May 22;12(7):e03739-23. doi: 10.1128/spectrum.03739-23 (PMC11324017; doi:10.1128/spectrum.03739-23)
Supplement: Supplemental tables — Tables S1-S4. [file spectrum.03739-23-s0002.docx]

**Table S1** AUCs at different dosing designs

|  | All (n=146) | Low-AUC group (n=107) | Intermediate-AUC group (n=25) | High-AUC group (n=14) | p value |
| --- | --- | --- | --- | --- | --- |
| AUC_0-24h_, µg h/mL | 421 (329, 497) | 391 (305, 486) | 470 (368, 555) | 451 (401, 539) | 0.009 |
| AUC_24-48h_, µg h/mL | 426 (357, 501) | 393 (319, 469) | 483 (421, 531) | 550 (436, 620) | <0.001 |
| AUC_ss_, µg h/mL | 440 (373, 535) | 411 (319, 496) | 518 (430, 593) | 617 (468, 665) | <0.001 |

Data are presented as medians (interquartile ranges). Statistical significance was set at p<0.05.

Abbreviations: AUC, area under the concentration-time curve. AUC_0-24h_, AUC on day 1; AUC_24-48h_, AUC on day 2; AUC_SS_, AUC at steady state.

**Table S2** AUC_24-48h_ values from the inverse estimation of the probability of AKI

| Percentage of AKI | Predicted of AUC_48h_ (95%CI) |
| --- | --- |
| All patients (n=146) |  |
| 5% | 318 (230–405) |
| 10% | 417 (355–478) |
| 15% | 478 (432–524) |
| 20% | 524 (487–561) |
| 25% | 562 (531–593) |
| Patients without TZP (n=121) |  |
| 5% | 340 (222–458) |
| 10% | 441 (359–522) |
| 15% | 504 (442–565) |
| 20% | 551 (502–599) |
| 25% | 590 (548–631) |

Abbreviations: AUC, area under the concentration-time curve; TZP, tazobactam/piperacillin. AUC_24-48h_, AUC on day 2.

**Table S3** Diagnostic accuracy of AKI factors (121 patients without TZP use)

|  | AUC | Cut-off value | Sensitivity | Specificity | PPV | NPV | Accuracy | TP | TN | FP | FN | p value |
| --- | --- | --- | --- | --- | --- | --- | --- | --- | --- | --- | --- | --- |
| AUC_24-48h_ | 0.84 | 400 | 1.00 | 0.47 | 0.20 | 1.00 | 0.53 | 14 | 50 | 57 | 0 | <0.001 |
|  |  | 462* | 0.93 | 0.68 | 0.28 | 0.99 | 0.71 | 13 | 73 | 34 | 1 |  |
|  |  | 500 | 0.71 | 0.78 | 0.29 | 0.95 | 0.77 | 10 | 83 | 24 | 4 |  |
|  |  | 600 | 0.29 | 0.94 | 0.40 | 0.91 | 0.87 | 4 | 101 | 6 | 10 |  |

Predictive performance of CART-derived and other candidate AUC toxicity thresholds.

Abbreviations: AKI, acute kidney injury; TZP, tazobactam/piperacillin; AUC, area under the concentration-time curve; PPV, positive predictive value; NPV, negative predictive value; TP, true positive; TN, true negative; FP, false positive; FN, false negative. * Optimal cutoff values.

**Table S4** Cox proportional hazard analyses of factors associated with AKI

|  | Univariate model | | | Multivariate model A | | | Multivariate model B | | |
| --- | --- | --- | --- | --- | --- | --- | --- | --- | --- |
|  | HR | (95% CI) | p value | HR | (95% CI) | p value | HR | (95% CI) | p value |
| Age, per 1-year increase | 1.0 | 0.98–1.05 | 0.405 |  |  |  |  |  |  |
| Sex; female | 0.8 | 0.33–2.00 | 0.652 |  |  |  |  |  |  |
| BMI, per 1 kg/m^2^ increase | 1.0 | 0.92–1.10 | 0.704 |  |  |  |  |  |  |
| SOFA score | 1.0 | 0.92–1.16 | 0.560 |  |  |  |  |  |  |
| APACHE II score | 1.0 | 0.91–1.12 | 0.912 |  |  |  |  |  |  |
| Sepsis | 1.0 | 0.41–2.45 | 0.997 | 0.92 | 0.36–2.31 | 0.854 |  |  |  |
| Septic shock | 1.8 | 0.72–4.51 | 0.212 |  |  |  |  |  |  |
| MRSA bacteremia | 1.3 | 0.38–4.64 | 0.648 |  |  |  | 0.52 | 0.14–1.97 | 0.339 |
| VAN AUC_24-48h_ |  |  |  |  |  |  |  |  |  |
| <500 µg·h/mL | Reference | - | - | Reference | - | - | Reference | - | - |
| 500-600 µg·h/mL | 3.7 | 1.29–10.58 | 0.015 | 4.5 | 1.51–13.61 | 0.007 | 5.3 | 1.69–16.60 | 0.004 |
| ≥600 µg·h/mL | 6.6 | 2.20–19.56 | <0.001 | 8.4 | 2.62–26.98 | <0.001 | 10.4 | 3.03–35.80 | <0.001 |
| Loding dose, ≥25mg/kg | 1.0 | 0.40–2.30 | 0.917 | 1.0 | 0.38–2.39 | 0.912 | 1.0 | 0.42–2.61 | 0.931 |
| Maintenance dose, mg/kg/day | 1.0 | 0.96–1.05 | 0.765 | 0.98 | 0.93–1.02 | 0.297 | 0.97 | 0.92–1.02 | 0.201 |
| eGFRcre, <30 mL/min/1.73 m^2^ | 0.6 | 0.08–4.37 | 0.601 |  |  |  |  |  |  |
| BUN/Scr, ≥20 | 1.2 | 0.38–3.46 | 0.799 |  |  |  |  |  |  |
| TZP | 2.1 | 0.79–5.40 | 0.136 |  |  |  |  |  |  |
| Catecholamine | 2.1 | 0.83–5.21 | 0.119 |  |  |  |  |  |  |
| Loop diuretic | 1.2 | 0.49–2.88 | 0.704 |  |  |  |  |  |  |

Statistical significance was set at p < 0.05. To account for the increased alpha error, the Bonferroni correction was applied to the comparison of the three groups, and a p-value of less than 0.0167 was considered significant. Abbreviations: AKI, acute kidney injury; HR, hazard ratio; CI, confidence interval; BMI, body mass index; SOFA, sequential organ failure assessment; APACHE II, acute physiology and chronic health evaluation II; VAN, vancomycin; AUC, area under the concentration-time curve; AUC24-48h, AUC on day 2; eGFRcre, creatinine-based estimated glomerular filtration rate; BUN/Scr, ratio of blood urea nitrogen to serum creatinine; TZP, tazobactam/piperacillin.
